# Predictors of everyday functional impairment in older patients with schizophrenia: A cross-sectional study

**DOI:** 10.3389/fpsyt.2022.1081620

**Published:** 2023-01-18

**Authors:** Chenxin Wu, Junrong Ye, Shaohua Li, Jialan Wu, Chen Wang, Lexin Yuan, Haoyun Wang, Yuanxin Pan, Xingxiao Huang, Xiaomei Zhong, Meilian Huang, Jianxiong Guo, Yuping Ning, Aixiang Xiao

**Affiliations:** ^1^Department of Geriatric Psychiatry, Affiliated Brain Hospital of Guangzhou Medical University (Guangzhou Huiai Hospital), Guangzhou, China; ^2^Department of Nursing, Affiliated Brain Hospital of Guangzhou Medical University (Guangzhou Huiai Hospital), Guangzhou, China; ^3^School of Nursing, Guangzhou Medical University, Guangzhou, China; ^4^Department of Nursing, The Third People’s Hospital of North Guangdong, Guangdong, China; ^5^Department of Chronic Diseases, Affiliated Brain Hospital of Guangzhou Medical University (Guangzhou Huiai Hospital), Guangzhou, China; ^6^Department of Office, Affiliated Brain Hospital of Guangzhou Medical University (Guangzhou Huiai Hospital), Guangzhou, China

**Keywords:** schizophrenia, older patients, everyday function, FAQ, cross-sectional study

## Abstract

**Objective:**

This study investigates the prevalence of everyday functional impairment among older adults with schizophrenia and builds a predictive model of functional decline.

**Methods:**

A total of 113 hospitalized older patients enrolled in this study. Functional impairment is defined according to the Functional Activities Questionnaire (FAQ). Patients who scored <9 could function independently daily, while those who scored ≥9 had problems in everyday functional activities. Data collected include sociodemographic characteristics, depressive symptoms, social support, and physical comorbidities, which were classified according to the eight anatomical systems of the human body.

**Results:**

The sample comprised 75% female participants with a mean age of 63.74 ± 7.42 years old. A total of 33.6% had a functional impairment, while cognitive impairment was present in 63.7%. Independent participants had better urinary system and respiratory system health (*P* < 0.05). After adjusting for the potential confounders of age, disease course, physical comorbidities, psychiatric symptoms, the ability to independently carry out daily activities, and cognitive function, we found that impaired everyday function is associated with poor cognition, depressive symptoms, first admission, psychiatric symptoms (especially positive symptoms), ADL, and respiratory and urinary system diseases.

**Conclusion:**

Everyday functional capacity is predicted by disease course, admission time, cognition, depressive symptoms, severity of psychosis, ability to carry out daily activities, and respiratory and urinary system health status. Urinary system diseases contribute significantly to the prediction of impaired function. Future studies should focus on health status, drug use, and everyday functional recovery in older patients with schizophrenia.

## Introduction

The number of senior citizens aged at least 60 years old in China has reached 264 million, or 18.7% of the country’s total population, according to data from the seventh national census in 2021 (1). There is increasing concern about the heavy burden of psychological disorders in the elderly population. Schizophrenia, a serious mental illness, has a lifetime prevalence of 0.9% in China and affects almost 1% of the population (2, 3). With higher rates of disability, recurrence, re-hospitalization, and mortality (4), schizophrenia exacerbates patients’ mental and physical conditions and places a heavy financial and care burden on the patients’ families and society.

Cognitive impairment is one of the core symptoms of schizophrenia, with almost all schizophrenia patients (98%) showing cognitive decline compared to their premorbid state (5). In addition, cognitive impairment may increase the risk of mild cognitive impairment (MCI) or developing dementia (4), which poses a significant threat to the diagnosis and rehabilitation of schizophrenia patients. Cognition impairment often manifests as impaired everyday function in the preliminary stage of schizophrenia, leading to problems carrying out the complex Instrumental Activities of Daily Living (IADL), which include managing finances and using public transportation. These deficits in everyday functioning may be further associated with problems in independent living, working, self-care, and socializing (6). Hence, everyday functioning is not only an important marker for screening cognitive alterations (7) but also a therapeutic target for rehabilitation (8).

The Functional Activities Questionnaire (FAQ) includes questions about basic daily functional activities, such as shopping, cooking, and taking buses, and is more sensitive to predicting cognitive scores than the IADL Scale (0.85 vs. 0.57) (9). Extensive research has explored the factors associated with functional impairment in dementia (10, 11). However, few studies have been conducted on the various functional determinants of older patients with schizophrenia.

Recent studies have confirmed that everyday functioning is strongly associated with cognitive performance in schizophrenia, particularly reflecting neurocognitive domains (12, 13). However, only a handful of studies have evaluated the functional capacity of schizophrenia patients with comorbidities. Alley et al. (14) reported that the incidence of functional impairment in obese individuals increased by 5.4% from 1998–1994 to 1999–2004 (from 36.8 to 42.2%, *P* = 0.03). Nonetheless, little is known about the impact of other comorbidities on everyday functioning, which hinders the process of full recovery in older patients with schizophrenia.

Therefore, this study aimed to explore the association between everyday functional capacity and current comorbidity in physical and mental conditions in elderly patients with schizophrenia. In line with previous studies, we hypothesized that the decline in functional activity in elderly patients with schizophrenia is associated with cognitive impairment (15). Second, risk factors of dysfunction in everyday instrumental activities were influenced by many physical and mental conditions, building a comprehensive predictive model involving diseases classified by the eight systems of the human body and other clinical outcomes.

## Materials and methods

### Participants

A retrospective cross-sectional survey was conducted between February 2021 and March 2022 in Guangdong, China. Participants included in this study were sampled consecutively from the geriatric psychiatric ward of a tertiary psychiatric hospital. The inclusion criteria were as follows: (1) old adults aged 50 years old and above (16); (2) hospitalized patients who were diagnosed with schizophrenia by psychiatrists based on DSM (Diagnostic and Statistical Manual of Mental Disorders)-IV criteria; (3) patients who could express and communicate with the investigators in general; and (4) patients who completed the informed consent and can complete questionnaires. Exclusion criteria were: (1) patients with severe somatic diseases; and (2) patients who were totally deaf or blind.

### Measurements

All outcomes were measured within 3 days from admission, including the patients’ sociodemographic characteristics, cognitive function, daily activity function, social support, nutrition status, and psychiatric symptoms. Patients’ demographic characteristics were collected using a self-made questionnaire which includes age, gender, marriage status, education, number of admissions, physical diseases, and so on. All investigators were trained for consistency and accuracy before the survey.

#### Mini-mental state examination

The Mini-mental State Examination (MMSE) is a screening tool that was developed by Folstein et al. (17) for assessing cognitive impairment in older adults. It includes five areas of cognitive function: orientation, registration, attention, calculation, and recall and language. The maximum score is 30 points (range 0–30), which is scored as the number of correctly completed items. Lower scores indicate greater cognitive impairment. MMSE scores <27 points are used to indicate impaired cognition, which has good validity and reliability in older adults (18).

#### Geriatric depression scale

The Geriatric Depression Scale (GDS) was specifically developed for detecting depressive symptoms in older people (19). The Chinese version of GDS-30 showed good reliability and validity (internal consistency of 0.89, intraclass correlation coefficient of 0.85). There are 30 items on the scale; each response in favor of depressive symptoms receives one point, while other responses are assigned zero points. The scores for the 30 items range between 0 and 30, and a cut-off value of 11 and above signifies the presence of depressive symptoms (20).

#### Brief psychiatric rating scale

The Brief Psychiatric Rating Scale (BPRS) scale used in China was designed and revised by Ming-yuan Zhang (21), which includes 18 questions in five areas: four items on anxiety and depression, four items on lack of vitality, four items on thought disorder, three items on activation, and three items on hostility. The BPRS is used to assess the severity and distribution of psychiatric symptoms. Each item is assessed on a Likert scale with seven coding levels ranging from 1 (absent) to 7 (very severe). The total score ranges from 18 to 126, with the cut-off point of mental disorder as 35.

#### Functional activities questionnaire

The FAQ was developed by Prefer in 1982 to assess the IADL in older adults (9). The Chinese version of the FAQ consists of 10 items: using tickets or paying bills on schedule; shopping alone for clothes, household necessities, and groceries; playing a game of skill; working on a hobby, doing chores, cooking, keeping track of current events, paying attention to, understanding, and discussing TV programs, books, and magazines; remember appointments, family occasions, and holidays; and traveling out of the neighborhood, driving, and arranging to take buses. Each item is rated on a three-point scale (0: normal; 1: requires assistance; 2: dependent). Total scores range from 0 to 30, with higher scores indicating less ability to perform daily activities. A score ≥9 indicates impairment of everyday functional activities and cognition.

#### Social support rating scale

The Social Support Rating Scale (SSRS) was developed by Xiao Shui-yuan in 1986 (22) and is widely used in China. It comprises 10 items in three dimensions: objective support, subjective support, and utilization of social support. The sum of each item represents its endpoint. SSRS scores <33 mean low social support, between 33 and 45 indicates moderate social support, and >45 is high social support. The reliability and validity were confirmed to be good, with Cronbach’s α ranging from 0.825 to 0.896 in older adults (23).

### Physical condition

#### Barthel index of activities of daily living

The Barthel Index of Activities of Daily Living (ADL) was developed by Mahoney in 1965 to assess patients’ dependence in terms of self-care and mobility. It is a quick and reliable assessment of fundamental ADL (24, 25). It includes ten items: feeding, bathing, grooming, dressing, bowels, bladder, toilet use, transfers (bed to chair and back), mobility (on level surfaces), and stairs. Total scores range from 0 to 100. A score ≤40 is classified as severely dependent and needs care from others completely, 41–60 is moderate dependency, whereby most activities need care from others; 61–99 indicates mild dependency, where few activities need care from others; 100 means independent and participants can take care of themselves completely.

#### Mini-nutritional assessment

The mini-nutritional Assessment (MNA) is a validated nutrition screening and assessment tool that can identify geriatric patients aged 65 and above who are malnourished or at risk of malnutrition. It has been revised into six questions and streamlines the screening process with good validity and accuracy. The maximum score is 14. A score of 12–14 points means normal nutritional status, 8–11 means risk of malnutrition, and 0–7 indicates malnourishment (26).

#### Statistical analyses

Data analysis was performed using SPSS statistical software version 20.0 (IBM Corp., Armonk, NY, United States). Patients were divided into two groups based on the FAQ cut-off point of 9. The original data from the measurement tools were normally or almost normally distributed, so they are presented as the mean and standard deviation (SD) were analyzed by student’s *t*-test. FAQ was presented as categorical variables. All categorical variables are expressed as frequencies and percentages. The Pearson Chi-squared test and Fisher’s exact test were used for categorical variables. Pairwise comparisons of statistically significant data from the Pearson Chi-squared test were performed using the *Z*-test, and the Bonferroni’s method was used to obtain the *p*-value. All variables with *P* < 0.05 in the univariate analyses were included in binary logistic regression models to identify the risk factors for impaired functional capacity. A two-tailed *p* < 0.05 was statistically significant.

## Results

### Baseline demographic characteristics and clinical outcomes

**FIGURE 1:**
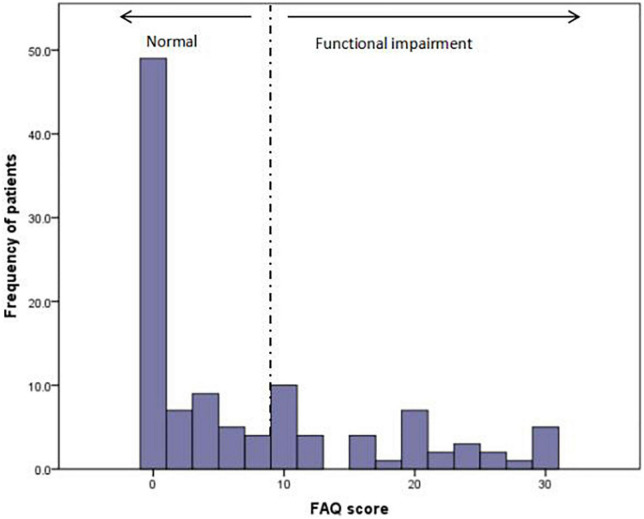
Distribution of functional impairment.

Elderly patients with schizophrenia were divided into two groups according to their FAQ scores. People who scored ≥9 points were classified as dependent or having functional impairment, while those who scored <9 were classified as normal or independent. The distribution of the FAQ scores is illustrated in Figure 1. Table 1 summarizes the dependent and independent patients’ demographic characteristics and selected clinical outcomes. The mean age of the participants was 63.74 ± 7.42 years old, ranging from 50 to 81. Of the 113 schizophrenia patients, 38 (33.6%) had impaired functional capacity. No significant differences were found regarding sex, age, education, marital status, or main caregivers. Compared with the normal group, elderly patients in the functional impairment group had a longer course of disease and higher incidences of respiratory and urinary diseases. As for mental symptoms, significantly lower GDS scores and psychiatric severity (BPRS) were observed in the independent group. Furthermore, cognitive function (MMSE) and abilities for daily activities (ADL) are better in the normal group.

**TABLE 1:** Comparison of demographic and clinical data between patients with dependent function capacity and independent function capacity.

Demographic characteristics	Coding	Total *N* = 113	Independent *N* = 75	Dependent *N* = 38	*X^2^/t*	*P*-value
		**Mean (SD) frequency (%)**	**Mean (SD) frequency (%)**	**Mean (SD) frequency (%)**		
Age		63.74 (7.42)	62.85 (7.02)	65.50 (7.96)	−1.810	0.073
Course of disease		18.52 (14.42)	16.37 (12.84)	22.78 (16.50)	−2.272	**0.025**
Gender	1 = Male	28 (24.78%)	19 (25.33%)	9 (23.68%)	0.037	0.848
	2 = Female	85 (75.22%)	56 (74.67%)	29 (76.32%)		
Education	1 = Primary or less	38 (33.63%)	27 (36.00%)	11 (28.95%)	0.562	0.453
	2 = Junior secondary school and above	75 (66.37%)	48 (64.00%)	27 (71.05%)		
Marital status	1 = Single (unmarried, divorced, death of a spouse)	34 (30.09%)	20 (26.67%)	14 (36.84%)	1.241	0.265
	2 = Couple (married)	79 (69.91%)	55 (73.33%)	24 (63.16%)		
Main caregivers	1 = None	10 (8.85%)	5 (6.67)	5 (13.16)	2.136	0.344
	2 = Nursing staff	6 (5.31%)	3 (4.00)	3 (7.89)		
	3 = Family member	97 (85.84%)	67 (89.33)	30 (78.95)		
First admission	1 = No	34 (30.09%)	17 (22.67)	17 (44.74)	5.840	**0.016**
	2 = Yes	79 (69.91%)	58 (77.33)	21 (55.26)		
BMI	1 = Underweight	14 (13.86%)	9 (13.04%)	5 (15.62%)	1.411	0.703
	2 = Normal weight	53 (52.48%)	35 (50.72%)	18 (56.25%)		
	3 = Overweight	27 (26.73%)	19 (27.54%)	8 (25.00%)		
	4 = Obesity	7 (6.93%)	6 (8.70%)	1 (3.12%)		
Payment method	1 = Self paid	14 (12.39%)	9 (12.00%)	5 (13.16%)	5.939	0.312
	2 = Basic medical insurance for urban residents	22 (19.47%)	12 (16.00%)	10 (26.32%)		
	3 = Basic medical insurance for urban workers	51 (45.13%)	33 (44.00%)	18 (47.37%)		
	4 = New cooperative medical care	1 (0.88%)	1 (1.33%)	0 (0.00%)		
	5 = Remote medical insurance	22 (19.47%)	17 (22.67%)	5 (13.16%)		
	6 = Covered by free medicare	3 (2.65%)	3 (4.00%)	0 (0.00%)		
SSRS		27.73 (5.37)	28.39 (5.45)	26.42 (5.03)	1.857	0.066
**Current comorbidity and symptoms**						
Circulatory system	1 = No	73 (64.60%)	51 (68.00%)	22 (57.89%)	1.126	0.289
	2 = Yes	40 (35.40%)	24 (32.00%)	16 (42.11%)		
Digestive system	1 = No	96 (84.96%)	65 (86.67%)	31 (81.58%)	0.511	0.475
	2 = Yes	17 (15.04%)	10 (13.33%)	7 (18.42%)		
Respiratory system	1 = No	85 (75.22%)	64 (85.33%)	21 (55.26%)	12.235	**<0.001**
	2 = Yes	28 (24.78%)	11 (14.67%)	17 (44.74%)		
Urinary system	1 = No	107 (94.69%)	74 (98.67%)	33 (86.84%)	7.014	**0.008**
	2 = Yes	6 (5.31%)	1 (1.33%)	5 (13.16%)		
Motor system	1 = No	103 (91.15%)	71 (94.67%)	32 (84.21%)	2.245	0.134
	2 = Yes	10 (8.85%)	4 (5.33%)	6 (15.79%)		
Endocrine system	1 = No	74 (65.49%)	51 (68.00%)	23 (60.53%)	0.623	0.430
	2 = Yes	39 (34.51%)	24 (32.00%)	15 (39.47%)		
Immune system	1 = No	105 (92.92%)	69 (92.00%)	36 (94.74%)	0.022	0.883
	2 = Yes	8 (7.08%)	6 (8.00%)	2 (5.26%)		
Neurological system	1 = No	86 (76.11%)	60 (80.00%)	26 (68.42%)	1.860	0.173
	2 = Yes	27 (23.89%)	15 (20.00%)	12 (31.58%)		
Reproductive system	1 = No	108 (95.58%)	72 (96.00%)	36 (94.74%)	0.093	0.761
	2 = Yes	5 (4.42%)	3 (4.00%)	2 (5.26%)		
BPRS		34.62 (6.72)	33.56 (6.34)	36.71 (7.03)	−2.405	**0.018**
GDS		9.46 (7.28)	7.92 (5.53)	12.50 (9.21)	−2.819	**0.007**
MMSE		22.50 (6.47)	24.05 (5.01)	19.42 (7.86)	3.310	**0.002**
Cognitive function level (MMSE)	1 = Normal	41 (36.28%)	32^a^ (42.67%)	9^b^ (23.68%)	15.544	**0.001**
	2 = Mild dementia	31 (27.43%)	23^a^ (30.67%)	8^a^ (21.05%)		
	3 = Moderate dementia	35 (30.97%)	20^a^ (26.67%)	15^a^ (39.47%)		
	4 = Severe dementia	6 (5.31%)	0 (0.00%)	6^a^ (15.79%)		
ADL		88.98 (16.97)	95.27 (9.79)	76.58 (21.02)	5.201	**<0.001**
MNA		9.15 (2.25)	9.44 (2.16)	8.58 (2.36)	1.943	0.055

BMI, body mass index; SSRS, social support rating scale; BPRS, brief psychiatric rating scale; GDS, geriatric depression scale; MMSE, mini-mental state examination; ADL, Barthel index of activities of daily living; MNA, the mini-nutritional assessment.

^a,b^Same letter means two groups has no difference statistically and vise versa. Bold values represent the *P* < 0.005, means they are statistical significant.

**FIGURE 2:**
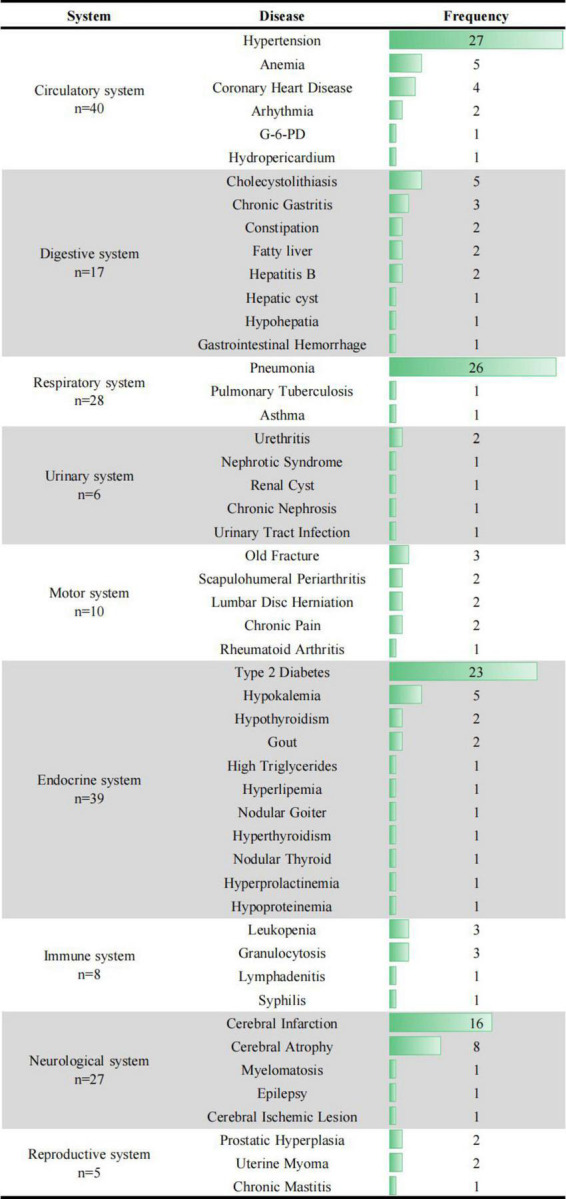
Frequency of diseases in different body systems.

Circulatory system (*n* = 40), endocrine system (*n* = 39), respiratory system (*n* = 28), neurological system (*n* = 27), and digestive systems (*n* = 17), were the most five common influenced body systems among recruited patients. In relation to different specified diseases, top 5 frequently diseases among recruited patients were hypertension (*n* = 27), pneumonia (*n* = 26), type 2 diabetes (*n* = 23), cerebral infarction (*n* = 16), and cerebral atrophy (*n* = 8), respectively (Figure 2).

### Variables associated with everyday functional capacity

Univariate analysis (Table 2) showed that disease course, first admission, respiratory system disease, urinary system disease, BPRS, GDS, MMSE, and ADL were significantly different between the normal and impaired function groups (*P* < 0.05). To reduce the effect of confounding factors, we further performed a multi-variable analysis with or without the occurrence of impaired function as a dependent variable (occurrence = 2, not occurrence = 1). Variables with *P* < 0.05 in the univariate analysis were included as independent variables.

**TABLE 2:** Binomial logistic regression showing the crude risk of dependent function capacity.

Variables	Coding	Univariate		
		**Unadjusted OR**	**(95% CI)**	***P*-value**
Age		1.051	(0.995–1.109)	0.073
Course of disease		1.032	(1.003–1.061)	**0.028**
Gender	1 = Male	(Reference)	(Reference)	
	2 = Female	1.093	(0.440–2.719)	0.848
Education	1 = Primary or less	(Reference)	(Reference)	
	2 = Junior secondary school and above	1.381	(0.593–3.214)	0.454
Marital status	1 = Single (unmarried, divorced, death of a spouse)	(Reference)	(Reference)	
	2 = Couple (married)	0.623	(0.271–1.436)	0.267
Main caregivers	1 = None	(Reference)	(Reference)	
	2 = Nursing staff	1.000	(0.132–7.570)	1.000
	3 = Family member	0.448	(0.121–1.663)	0.230
First admission	1 = No	(Reference)	(Reference)	
	2 = Yes	0.362	(0.157–0.836)	**0.017**
BMI	1 = Underweight	0.926	(0.270–3.174)	0.902
	2 = Normal weight	0.758	(0.193–2.983)	0.692
	3 = Overweight	0.300	(0.028–3.250)	0.322
	4 = Obesity	0.930	(0.861–1.006)	0.069
SSRS				
**Current comorbidity and symptoms**				
Circulatory system	1 = No	(Reference)	(Reference)	
	2 = Yes	1.545	(0.690–3.461)	0.290
Digestive system	1 = No	(Reference)	(Reference)	
	2 = Yes	1.468	(0.510–4.221)	0.476
Respiratory system	1 = No	(Reference)	(Reference)	
	2 = Yes	4.710	(1.906–11.637)	**0.001**
Urinary system	1 = No	(Reference)	(Reference)	
	2 = Yes	11.212	(1.260–99.771)	**0.030**
Motor system	1 = No	(Reference)	(Reference)	
	2 = Yes	3.328	(0.878–12.612)	0.077
Endocrine system	1 = No	(Reference)	(Reference)	
	2 = Yes	1.386	(0.616–3.120)	0.431
Immune system	1 = No	(Reference)	(Reference)	
	2 = Yes	0.639	(0.123–3.328)	0.595
Neurological system	1 = No	(Reference)	(Reference)	
	2 = Yes	1.846	(0.760–4.485)	0.176
Reproductive system	1 = No	(Reference)	(Reference)	
	2 = Yes	1.333	(0.213–8.340)	0.758
BPRS		1.076	(1.011–1.144)	**0.021**
GDS		1.091	(1.031–1.155)	**0.003**
MMSE		0.891	(0.834–0.953)	**0.001**
ADL		0.921	(0.890–0.954)	**<0.001**
MNA		0.840	(0.702–1.006)	0.058

BMI, body mass index; SSRS, social support rating scale; BPRS, brief psychiatric rating scale; GDS, geriatric depression scale; MMSE, mini-mental state examination; ADL, Barthel index of activities of daily living; MNA, the mini-nutritional assessment. Bold values represent the *P* < 0.005, means they are statistical significant.

Binary logistic regression analyses showed that cognitive function (OR: 0.873, 95% CI: 0.783–0.974, *P* = 0.015), ability for daily activities (OR: 0.948, 95% CI: 0.907–0.991, *P* = 0.019) are protective factors in this study; depression (OR: 1.144, 95% CI: 1.052–1.245, *P* = 0.002), respiratory system disease (OR: 6.950, 95% CI: 1.670–28.930, *P* = 0.008), and urinary system disease (OR: 30.323, 95% CI: 1.296–709.427, *P* = 0.034) were independent risk factors for functional impairment in older schizophrenia patients with adjustment for other covariates with *P* < 0.05 in the univariable analysis. The detailed results of the logistic analysis are shown in Table 3.

**TABLE 3:** Results for binomial logistic regression, displaying adjusted odds ratios and 95% confidence intervals for functional impairment.

Variables coding		Multivariate*		
		**Adjusted OR**	**(95% CI)**	***P*-value**
Course of disease		0.999	(0.959–1.041)	0.977
First admission	1 = No	(Reference)	(Reference)	
	2 = Yes	0.307	(0.088–1.071)	0.064
Respiratory system	1 = No	(Reference)	(Reference)	
	2 = Yes	6.950	(1.670–28.930)	**0.008**
Urinary system	1 = No	(Reference)	(Reference)	
	2 = Yes	30.323	(1.296–709.427)	**0.034**
BPRS		1.062	(0.976–1.155)	0.166
GDS		1.144	(1.052–1.245)	**0.002**
MMSE		0.873	(0.783–0.974)	**0.015**
ADL		0.948	(0.907–0.991)	**0.019**

*Adjusted with *P* < 0.05 in univariable analysis course of disease, first admission, respiratory system diseases, urinary system diseases, BPRS, GDS, MMSE, and ADL. Bold values represent the *P* < 0.005, means they are statistical significant.

The probability test outcome variable for predicting impaired function was generated based on a multi-variable logistic regression data model, and it was plotted as a ROC curve to predict deficit function outcomes. The area under the ROC curve was 0.901, showing that the model had a good predictive value (*P* < 0.001, 95% CI: 0.841–0.960).

## Discussion

The objective of this study was to identify factors associated with everyday functional impairment in older adults with schizophrenia. We found that the prevalence of functional impairment is moderately high in elderly patients with schizophrenia. The risk factors for dysfunction were influenced by several factors. After adjusting for confounding factors, we found that better cognitive function and abilities for daily activities significantly reduced the incidence of functional dependency. While being accompanied by depressive symptoms, respiratory and urinary systems disease pose a great threat to developing functional impairment.

Consistent with previous studies, we found that dependence on everyday tasks was related to cognitive function and ability for daily activities (6). People with independent social function were found to have a better cognitive function and daily abilities in our study. In our study, we found urinary tract infection, urethritis, nephrotic syndrome, and chronic kidney disease, were prevalent urinary system diseases among aged schizophrenia patients. Current research shows that most antipsychotic drugs, such as risperidone, sulpiride, clozapine, and perphenazine, are metabolized by the liver and then excreted by the kidney after being ingested by the human body. Long-term use of these antipsychotic drugs can cause varying damage to the liver and kidney (27). Thus, the impaired urinary function may be indirectly induced by the impact of long-term antipsychotic drug use. Further research is needed about drug administration.

In regard to respiratory diseases, we also noticed pneumonia was the most prevalent disease among aged schizophrenia patients, approximately 23.0% (26/113) of them were diagnosed with after admission. Davydow et al. noted pneumonia in older adults was associated with subsequent functional and cognitive impairment, because individuals with pneumonia showed lower levels of aerobic capacity (28, 29). Further study by Holmen et al. (30) investigated 80 patients of schizophrenia, and pointed that cardio-respiratory fitness could explained 9.1% of the variance in state-sensitive cognitive functioning (30). Therefore, respiratory functioning is one of the most significant factors influencing functional impairment of daily activities among aged schizophrenia patients.

In addition, what we found interesting is that the psychiatric symptoms (BPRS score) and the course of disease were found to be risk factors in univariate analysis, but both did not present statistical significance in the multivariate analysis. However, this is not inconsistent with previous findings; extensive cross-sectional studies have shown that negative symptoms, such as anhedonia and depressive symptoms, are correlated with an everyday function instead of positive psychotic symptoms in schizophrenia (13, 31). Following factors accounting for the difference of results among studies should be noticed: first, psychiatric symptoms and disease duration may indirectly correlate with functional impairment; second, the sample size in this study may be too small to create a robust model.

This study indicates that identifying everyday social function is an easy and important way of detecting cognitive impairment in schizophrenia patients. Because of the potential impact of different comorbidities associated with functional capacity in older adults with schizophrenia, the results of this study can expand the existing data and provide scientific direction for subsequent strategies for preventing and improving everyday functional impairment.

Although important discoveries have been revealed in this study, limitations should be noted. In general, this study investigated the functional impairment of schizophrenia, and examined the diseases that might influence functional impairment according to different human body systems. In data analysis, only the outcomes of human body system were set as covariates, therefore further large-sample studies are needed to explore the correlation of daily functional impairment and different diseases. Additionally, since Guangzhou is a relatively well-developed area in southern China, data from a tertiary, public psychiatric hospital might influence the national generality of our conclusion, thus our findings should be interpreted with cautions.

## Conclusion

This study showed that everyday functional capacity is associated with physical diseases. Respiratory system diseases (such as pneumonia and bronchitis) and urinary system diseases (such as urinary tract infection and nephrotic syndrome) could worsen patients’ everyday functioning. Mood status, especially depression, cognition, and ADL, might determine everyday function impairment rather than psychopathic symptoms. In short, everyday function impairment reflects various aspects of physical and mental health in elderly schizophrenic patients. Our results recommend that great attention should be paid to improving the ability for daily activities and treating accompanying depression or respiratory and urinary system diseases. Everyday function decline may be a sign of cognitive impairment. Further research needs to track the characteristics of dynamic changes in everyday functional capacity.

## Data availability statement

The raw data supporting the conclusions of this article will be made available by the authors, without undue reservation.

## Ethics statement

The studies involving human participants were reviewed and approved by IRB, The Affiliated Brain Hospital of Guangzhou Medical. The patients/participants provided their written informed consent to participate in this study.

## Author contributions

JY, XZ, and AX conceived the original idea of this study. CWu, JW, CWa, HW, YP, SL and LY participated in data collection and process, any differences in determinations of inclusion/exclusion or study quality existed between them were discussed. CWu, JY and SL drafted the manuscript, AX, JG, YN, YP, CWu and JY amended the manuscript. SL, XH, and MH also participated in data statistical analysis. All authors read and approved the final manuscript. CWu, JY and SL contributed equally to this study, and were assigned to be co-first authors. AX, YN and JG were assigned to be correspondence. All authors contributed to the article and approved the submitted version.
